# Givinostat, a type II histone deacetylase inhibitor, induces potent caspase-dependent apoptosis in human lymphoblastic leukemia

**DOI:** 10.18632/genesandcancer.117

**Published:** 2016-09

**Authors:** Ying Li, Kevin Zhao, Chenjiao Yao, Samir Kahwash, Yan Tang, Guojiuan Zhang, Kara Patterson, Qi-En Wang, Weiqiang Zhao

**Affiliations:** ^1^ The Third Xiangya Hospital of Central South University, Hunan, China; ^2^ Department of Pathology, The Ohio State University Wexner Medical Center, Columbus, OH, USA; ^3^ Department of Pathology, Nationwide Children's Hospital, Columbus, OH, USA; ^4^ Department of Radiology, The Ohio State University Wexner Medical Center, Columbus, OH, USA

**Keywords:** acute lymphoblastic leukemia, givinostat, apoptosis, p53, BCR-ABL

## Abstract

Unlike chronic myeloid leukemia, patients with acute lymphoblastic leukemia (ALL) with Philadelphia chromosome (*Ph+*) do not respond well to Imatinib or tyrosine kinase inhibitors (TKI). In addition, TKI might induce resistant mutations in kinase domain (KD) of *ABL* in patients with relapsed diseases. Of the histone deacetylase (HDAC) inhibitors, suberoylanilide hydroxamic acid (SAHA) has shown to induce potent cytotoxicity on acute myeloid leukemia cell lines but Givinostat effect on acute lymphoblastic leukemia (ALL) has not been reported. We investigated if Givinostat could exert similar inhibitory effect on SUP-B15, an established B-cell ALL with Philadelphia chromosome (*Ph+*). Two *Ph*+ leukemia cell lines, SUP-B15 and an AML cell line K562 were studied in parallel for their responses to Givinostat. Mutation status of *TP53* genes was also examined to correlate cellular proliferation and apoptosis. Givinostat significantly inhibited cell proliferation of SUP-B15 (IC_50_:0.18±0.03μM) and simultaneously inhibited BCR-ABL signal pathway. A remarkable apoptosis was induced by 0.25μM Givinostat in SUP-B15 along with the activation of caspase cascades and increased expression of p21. These inhibitory and proapoptotic effects were not observed in K562 simultaneously treated with Givinostat. Finally our studies showed that *TP53* mutation status might determine responder or non-responder to Givinostat in these two *Ph+* leukemia cell lines.

## INTRODUCTION

The *BCR-ABL1* fusion genes resulted from the translocation of t(9;22)(q34;q11) or Philadelphia Chromosome (*Ph+*) are found in virtually all chronic myelogenous leukemia (CML), one third of adult lymphoblastic leukemia (ALL), and occasionally in acute myeloid leukemia (AML) [1-2]. The chimeric BCR-ABL proteins constitutively possess tyrosine kinase activities which are postulated to be responsible for the development of leukemia via activating the Ras and mitogen-activated protein kinase pathway (RAS-MAPK), Janus-kinase (JAK)-signal transducer and activator of transcription pathways (JAK-STAT), and bcl-2/Bad/Bcl-xL anti-apoptosis signal pathway to promote cell proliferation, antiapoptosis, and genomic instability [1-3]. Tyrosine kinase inhibitors (TKI) for BCR-ABL, such as Imatinib and Dasatinib, have achieved great success in treatment of CML [4-5]. One of the mechanisms of TKIs induced apoptosis in K562 AML cells is proposed by trapping BCR-ABL in the nuclei of leukemic cells [6]. In contrast, patients with *Ph+* ALL do not respond well to these target medicines [7]. In addition, a previous study demonstrated that TKI might induce resistant tyrosine kinase domain (KD) mutations in *ABL* in the vast majority of patients with recurrent disease that received TKI therapy [8]. Therefore, efforts on finding novel therapeutic agents and approaches will benefit these patients.

Acetylation and deacetylation of N-terminal tails of histones regulated by histone acetyltransferases or histone deacetylases (HDACs) result in remolding of chromatin which selectively turn on or off the genes of the interest, hence being ideal epigenetic targets by medicine, namely HDAC inhibitors [9-11]. Suberoylanilide Hydroxamic Acid (SAHA) also known as Vorinostat is a prototype of HDAC inhibitor in the treatment of both solid and hematologic malignancies [12-13]. Givinostat (ITF2357), similar to SAHA with combined Class I+II HDAC inhibitory effects has shown anti-inflammatory properties at low nanomolar concentrations in humans, and proven to be a safe oral medication [14]. Previous study showed its anti-neoplastic activities against cells with *JAK2* V617F mutation, a hallmark for human myeloproliferative neoplasm (MPN) [15-17], and on a T-cell ALL [18]. In this study we demonstrated that Givinostat also had potent anti-leukemic effects in SUP-B15, a *Ph*+ B-cell ALL cell line resistant to TKIs.

## RESULTS

### Givinostat induced anti-proliferation of ALL cells and inhibited BCR-ABL signal pathway

**Figure 1:**
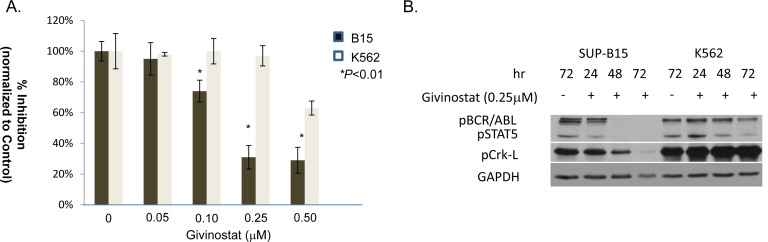
Antiproliferation effect of Givinostat on leukemia cell lines **A.** The growth inhibition of SUP-B15 and K562 leukemia cells by Givinostat. The viabilities of cultured cells were determined by MTT assay (see Materials and methods) with Givinostat from 0.05 to 0.50μM at 48hrs of incubations. Data were represented as mean±SD from at least three repeats. The asterisk represents significantly lower values as compared with control (*P < 0.01). **B.** Western blotting of cultured cells treated with or without Givinostat (0.25μM) to reveal inhibitory effects on the BCR-ABL signal pathways in *Ph+* leukemia cells. MTT, methylthiazol tetrazolium.

We performed dose-effect study of Givinostat at 48hrs on K562 and SUP-B15. Givinostat significantly suppressed the proliferation of SUP-B15 starting at 0.10 μM and reached to a plateau at 0.25 to 0.50μM. Givinostat had minimum inhibition on K562 except at highest concentration tested, 0.5μM (Figure 1A). The IC_50_ of Givinostat treatment at 48 hours was determined from cell survival plots (SigmaPlot). The IC_50_ of Givinostat on SUP-B15 was 0.18±0.03μM while on K562 was 4.6±0.35μM, and the difference was statistically significant (*P* < 0.0039, *n* = 3).

The activities in BCR-ABL signal pathway in leukemia cells treated by 0.25μM Givinostat were studied at various post-treatment time points and demonstrated in Western blots (Figure 1B). Obvious and significant reductions of all three key phosphoproteins in BCR-ABL signal pathways were observed in SUP-B15 beginning at 24 hours. The pBCR-ABL and pSTAT5 were virtually entirely lost at 48hr and pCrkL totally lost at 72hr. On the contrary, no inhibitory effects of Givinostat were observed on pBCR-ABL and pCrkL except pSTAT5 which were lowered at 48 and 72hrs in K562. The pCrkL protein in K562 was even slightly more at 72 hours in treated than the controls.

The inhibitory effect of Imatinib at concentrations from 0.5 to 5μM on SUP-B15 and K562 were studied (Data not shown). At 0.5μM, Imatinib inhibited about 44% (±5.0) of K562 cell growth at 48 hours, and barely inhibited SUP-B15 (16%±4.0), hence was significantly less effective than K562 (*P* < 0.0001) and consistent with previous finding (Quentmeier et al, 2011) [7].

### Givinostat induces potent apoptosis in Pre-B ALL cells

**Figure 2:**
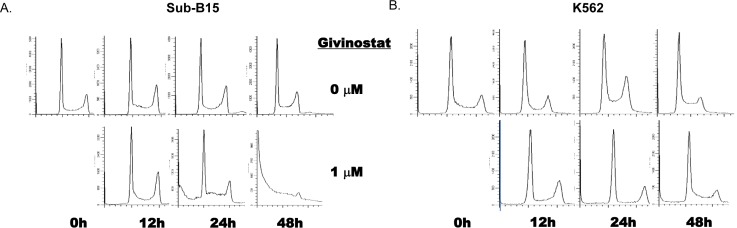
Induction of apoptosis by Givinostat on leukemia cell lines The induction of apoptosis measured by sub-G0/G1 fractions by flow cytometry in cells of **A.** SUP-B15 and **B.** K562 treated with Givinostat (1mM). The cultured cells were stained with PI (see Materials and methods) at 0 to 48hrs of incubation with Givinostat. Data were one of three representative tests (Detailed data were summarized in Table 1). PI, Propidium Iodine. Fractions were analyzed using software Mod-Fit.

We examined the effect of Givinostat on cell viability using both cell cycle analysis and Annexin V PI assay by flow cytometry. Givinostat at 1.0μM exhibited strongly cytotoxicity activities in SUP-B15 as evidenced by significant increases of sub-G0/G1 (apoptotic/ necrotic) fractions in 24 to 48hrs (Figure 2A and Table 1). Givinostat exhibited a much less cytotoxic effect on K562. The sub-G0/G1 fractions of SUP-B15 were 37.6±5.4% and 89.9±1.9% at 24hrs and 48hrs, respectively, which were statistically significantly higher than those in K562, 18.1±3.1% and 27.8±12.8% at the same time period (*P* < 0.05 and *P* < 0.01 respectively).

**Table 1:** Differences in Givinostat-induced cytotoxicity (Sub-G0/G1 fractions) in leukemia cells quantitatively analyzed by flow cytometry*

Time (hr)	Sup-B15	K562	*P*-value
0	4.81±2.4	6.9±2.7	0.340
12	6.4±3.2	2.81±0.9	0.175
24	37.6±5.4	18.1±3.1	0.041
48	89.9±1.9	27.8±12.8	0.010

* The data was presented as Mean ± S.D. of at least three experiments. Both cells exposed to 1.0μM Givinostat from 12, 24 and 48 hrs. Flow cytometric analysis was performed as described in Materials and Methods.

**Figure 3:**
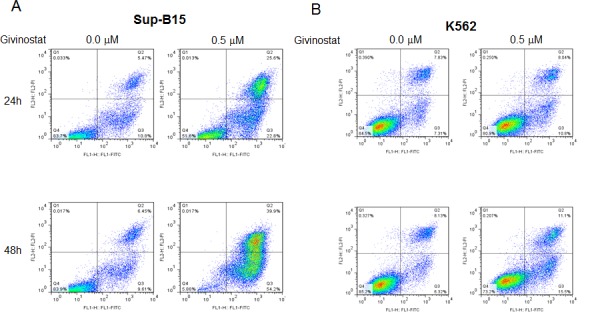
Pro-apoptosis of Givinostat on leukemia cells The induction of apoptosis measured by V-FITC/PI fractions by flow cytometry in cells of **A.** SUP-B15 and **B.** K562 treated with Givinostat (0.5μM). The cultured cells were stained with 5μl of FITC Annexin V and 5μl of PI (see Materials and methods) at 24 and 48hrs of incubation with Givinostat. Data were one of three representative tests (Detailed data were summarized in Table 2). PI, Propidium Iodine. Fractions were analyzed using software FlowJo software (TreeStar).

Since Sub-G0/G1 fractions comprised of both apoptotic and necrotic cells, we used more accurate approach by using Annexin V-FITC/PI and quantitated by flow cytometric analysis (Figure 3) to quantify the apoptosis. With reduced Givinostat, 0.5μM, apoptosis in SUP-B15 was induced to 54% to 92.9% at the posttreatment of 24hrs to 48hrs. On the opposite hand, only 13.4% to 9.8% of K562 cells underwent apoptosis at the same doses, and differences were statistically significant when compared to SUP-B15 (*P* < 0.001).

**Table 2:** Differences in Givinostat-induced apoptosis in leukemia cells detected by FITC-Annexin V/PI and quantitatively analyzed by flow cytometry^#^

Leukemic cells	Time (hr)	Viable (%)	Early Ap (%)	Late Ap (%)	Necrotic (%)
B15	24	46.1±4.92**	22.6±0.9**	31.4±5.4*	0.02±0.01
K562		86.0±1.8	7.7±1.6	5.7±0.8	0.3±0.1
B15	48	7.1±3.6**	57.5±5.0**	35.4±1.4*	0.02±0.01
K562		77.2±2.2	6.7±1.4*	5.1±0.9	0.3±0.1

# Mean ± S.D. of at least three experiments. Both cells exposed to 0.25 μM Givinostat for 24 or 48 hrs. Flow cytometric analysis was performed as described in Materials and Methods.

* *P* < 0.001

** *P* < 0.05. Ap: apoptosis

**Figure 4:**
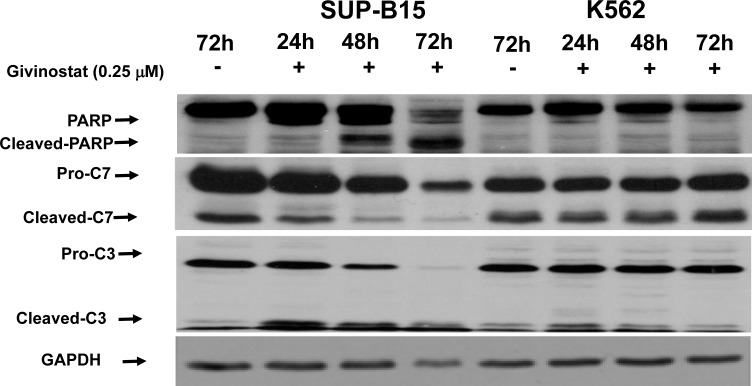
Activation of apoptotic cascade by Givinostat on leukemia cell lines Western blotting of cultured cells treated with or without Givinostat (0.25μM) to reveal activation of apoptotic cascade in *Ph+* leukemia cells. The data represents one of three repeats. The cleaved forms of caspase-3 were at 17 kDa, caspase-7 at 20 kDa, and PARP1 at 85 KDa. PARP1, Poly (ADP-Ribose) Polymerase 1; C7 Caspase 7; C3: Caspase 3; GAPDH: Glyceraldehyde-3-Phosphate Dehydrogenase.

The mechanisms of Givinostat-induced apoptosis in SUP-B15 were further studied by a Western blot to evaluate the status of caspase cascades. As shown in Figure 4 (SUP-B15, left panel), cleavages of caspase-3, −7 and PARP1 were detected at 24hrs and maximized at 72hrs after treatment in SUP-B15. On the contrary, except intrinsic background levels in caspase-3 and −7, all these apoptotic proteins were intact in K562 (K562, Figure 4). These data suggested that apoptosis induced by Givinostat in SUP-B15 is caspase-dependent and caspase-mediated PARP1 cleavage occurred upon caspase-activation.

### The apoptosis induced by Givinostat might be p53-dependent

**Figure 5:**
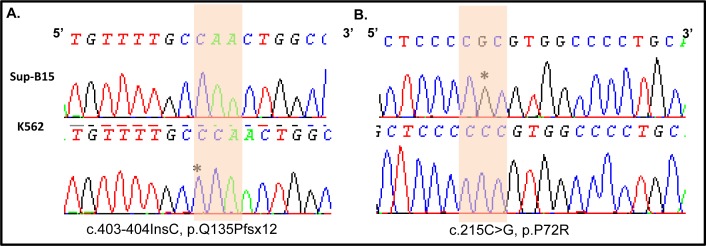
Verification of TP53 gene mutations in leukemia cell lines Traces of Sanger sequencing was shown **A.** an area of exon 5 showing homozygous mutations of c.403-404insC or p.Q135Pfsx12 in of K562 (lower panel). SUP-B15 shows a wild-type (upper panel). *pinpoints the inserted cytidine. **B.** an area of exon 4 of *TP53* with a homozygous mutation, c.213C>G, p.P72R in SUP-B15 (upper panel). *indicates the mutated Guanine with no change in K562 (Lower panel). Shaded areas indicate the mutated codons.

Apoptosis can occur via both extrinsic and intrinsic pathways and the mechanisms of Givinostat induced apoptosis in ALL cells are still unknown. In response to cellular stress, p53 mediates apoptosis through a linear pathway involving bax/cytochrome c/caspase-9 activation, followed by the activation of caspase-3, −6, and −7 cascades. Since capase-3, −7 and PARP1 activations were confirmed in Givinostat-induced apoptosis in SUP-B15 but not in K562, we postulate that p53 is functional and *TP53* gene is not mutated in SUP-B15. *TP53* in K562 is known in null (*TP53*−/−) status due to a homozygous frameshift mutation [21], and will provide an excellent cell model to confirm our postulation. Since K562 cell line has passed numerous passages since its establishment, it is critical to further confirm that K562 maintained in our laboratory still had the authentic *TP53*−/− status. Therefore, we reexamined the mutation status of *TP53* in K562 along with SUP-B15. Using high resolution melting (HRM) technology (Materials and Methods) we first screened all of 11 exons of *TP53*. The mutations were then confirmed by direct DNA Sanger sequencing. In our study, the homozygous c.403_404insC in exon 5 of *TP53* in K562 was confirmed by HRM and Sanger sequencing (Figure 5, right panel), which is predicted to have a nonfunctional truncate protein (p.Q135Pfsx12). In SUP-B15, only a single synonymous homozygous mutation, c.213C>G (p.P72R), in exon 4 of *TP53* (Figure 5, left panel) was found.

**Figure 6:**
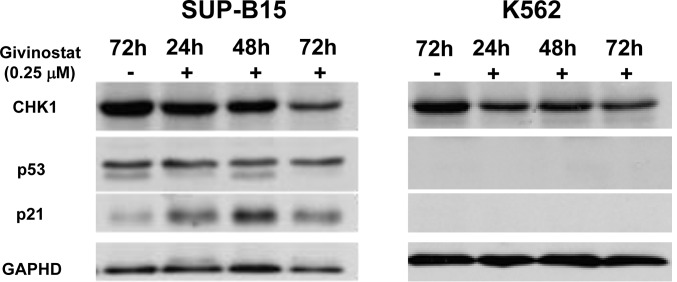
Detection of expressions of CHK1, p53, and p21 in leukemia cells Western blotting of cultured cells treated with or without Givinostat (0.25μM) to reveal p53 integrality in SUP-B15 (left) and K562 (right) at 24 to 72hrs. GAPDH: Glyceraldehyde-3-Phosphate Dehydrogenase.

Protein expressions of p53 and CHK1 and p21 were further analyzed by Western blot assay in cells treated with or without 0.25μM Givinostat. As shown in Figure 6 (left panel), while no significant changes in expressions of p53 and CHK1 in SUP-B15 from 24 to 72 hours, the expressions of p21, however, were elevated at 24 and 48 hours, but reduced at 72 hours as compared to untreated SUP-B15. On the contrary p53 and p21 proteins in K562 were undetectable which is consistent with the *TP53*−/− status. The CHK1 expression was unaffected in K562 and independent of p53/p21activities in K562.

## DISCUSSION

In this study, our data confirmed that Givinostat, a Class I and II HDAC inhibitor, is a potent inhibitor for cellular proliferation and strong inducer for apoptosis in Imatinib-resistant *Ph*+ B-cell lymphoblastic leukemia cells, SUP-B15. This observation provides the first demonstration, to our knowledge, that apoptosis in *Ph+* ALL leukemia cells induced by Givinostat requires intact p53 and is caspase-dependent.

The oncogenic protein, BCR-ABL, has a constitutively active tyrosine kinase which drives cellular proliferation and anti-apoptosis [1-3]. Though Imatinib and other TKIs are potent inhibitors for CML, *Ph+* B-ALL patients or established cell lines from these patients were resistant to these conventional TKIs, most likely due to high and constant expressions of phosphorylated BCR-ABL and its substrate proteins, STAT5 and CrkL, or partly due to Imatinib-induced T351I mutation in *ABL* domain [8. 22]. In this study, *in vitro* treatment of lymphoblastic leukemia cells with Givinostat directly inhibited BCR-ABL signal pathway with significant loss of key phosphoproteins of pBCR-ABL, and pSTAT5 and pCrkL (Figure 1B) in a similar pattern observed in Imatinib-treated CML and K562 [11, 23], but in different mechanisms. Previous studies have shown that the HDAC inhibitor might enhance degradation of BCR-ABL proteins secondary to hyperacetylation of the chaperon protein, HSP90 [24-26]. This hypothesis, however, cannot explain why inhibition on K562 by Givinostat was not observed in this study.

We studied induction of apoptosis of Givinostat on these two cell lines in hope to find the answers. Again, the apoptosis was prominently induced in SUP-B15 but not in K562. Previous study showed, though with limited numbers, that one of most important contributions to treatment failure in children and adult ALL is the presence of mutations/deletions of *TP53* gene among these patients [27-28]. The major anti-neoplastic function of p53 is to arrest the cells at G1 phase followed by initiating apoptosis through induction of p21 and PUMA, its transcriptional targets, as observed in IR-treated cells [29]. In this study, we confirmed *TP53* gene in SUP-B15 only has a homozygous p.P72R mutation, which doesn't affect p53 expression as shown in Figure 6. First identified by Ara et al. (1990) [30], p.P72R mutation is considered as a synonymous benign mutation though Dumont et al. (2003) found that R72 had up to 15-fold increased apoptotic ability compared with P72 in both inducible Saos2 (a human osteosarcoma) cell lines and H1299 (a human lung adenocarcinoma) cells [31]. Finally p53 protein can be stabilized by the hyperacetylation of p53 [32]. Therefore, p53 might have induced p21 and its signal pathway resulting in cell cycle arrest and apoptosis in leukemia cells when the oncogenic driven factors, pBCR-ABL/pSTAT5/pCrkL, were removed by Givinostat.

On the contrary, no p53 is detectable in K562 due to the null mutation (c.403_404insC). Again these cells with defect p53 fail to neither induce p21 expression nor activate the apoptotic cascade including absent cleavage of PARP1, a sensitive marker for apoptosis at later stage.

We demonstrated in this study that *in vitro* treatment with a single HDAC inhibitor, Givinostat, resulted in significant inhibition on cell proliferation and induction of apoptosis in a *Ph*+ Pre-B ALL cell line. The anti-leukemic effect of Givinostat on *Ph+* B-cell leukemia might depend on intact p53/p21. Our data strongly suggests that Givinostat can function as a potent and ideal anti-leukemic candidate drug among patients with *Ph+* pre-B ALLs. A screen for *TP53* mutation might be needed if it is considered to apply clinically. More studies like *in vivo* model to assess the therapeutic effects of Givinostat in patients-derived xenograft or clinical trials might help translate this study to clinical utilizations.

## MATERIALS AND METHODS

### Reagents and cell culture

Iscove's Modified Dulbecco's Medium (IMDM), heat-activated fetal bovine serum (FBS), and antibiotic/antifungal reagents were purchased from Life technologies (Grand Island, NY). Leukemia cell lines, K562 (AML) and SUP-B15 (ALL), were obtained from American Type Culture Collection (ATCC, Rockville, MD), and maintained in IMDM supplemented with 0.5X antibiotic/ antifungal reagents and 10 to 20% FBS. The methylthiazol tetrazolium reduction (MTT) testing kit was purchased from Promega (Madison, WI). Givinostat (C24H27N3O4, 421.489 g/mol) and Imatinib (C29H31N7O, 493.603 g/ mol) were purchased from Selleck Chemicals (Houston, TX), and prepared per instruction pamphlet. Antibodies used in Western blots for detection of Caspase 3 and 7, pBCR-ABL, pStat5, and pCrkL were purchased from Cell Signaling (Danvers, MA). The antibodies for detection of Checkpoint kinase 1 (CHK1), cyclin-dependent kinase inhibitor 1 (p21, or p21Cip1, or p21Waf1), tumor protein p53 (p53, DO-1), Poly (ADP-Ribose) Polymerase 1 (PARP1) and Glyceraldehyde-3-Phosphate Dehydrogenase (GAPDH) were purchased from Santa Cruz Biotechnology (Santa Cruz, CA). Phosphate-buffered saline (PBS) and Radioimmunoprecipitation (RIPA) lysis buffer were purchased from Santa Cruz. The Annexin V-fluorescein isothiocyanate (FITC) kit for detection of apoptosis by flow cytometry was purchased from BD Biosciences (San Jose, CA), and Propidium Iodide (PI) from Life technologies (Grand Island, NY). Dimethyl sulfoxide (DMSO) was purchased from Sigma (St. Louis, Missouri). Cells were treated with Givinostat or Imatinib in different concentrations (0.1-1mM) for intervals (12-72hr). Cell proliferation was assessed by the MTT test as described previously [19].

### Immunoblotting

Freshly cultured cells at 2.0×107 with or without treatment were harvested, washed with PBS, and re-suspended in RIPA lysis buffer containing proteinase and phosphatase inhibitors. Protein concentration was determined using the Bio-Rad protein assay (Bio-Rad, Hercules, CA). Sixty (60) μg of protein was separated on SDS-PAGE and transferred to polyvinylidene difluoride (PVDF) membranes, probed with antibodies. The blots were visualized with ECL reagent (Amersham, Arlington Heights, IL) and then exposed to autoradiography film (Denville, Metuchen, NJ). All tests were repeated three times.

### Flow cytometry analysis

Freshly cultured cells at 2.0×106 with or without treatment were washed with 1xPBS, and suspended in Propidium Iodine (PI) (0.02mg/ml)/Triton X-100 (0.1%)/ PBS solution with RNase A (0.2mg/ml) and stained for 1hr at room temperature (20°C). Cells were analyzed using a flow cytometry (FACSCalliber, BD Bioscience, San Jose, CA) and data analyzed for cell cycle distribution (Mod-Fit software). For flow cytometric analysis for apoptosis using Annexin V-FITC/PI kit (BD Biosciences, San Jose, CA), 1×106 freshly cultured cells were harvested, washed with 1xPBS, re-suspend in 1x binding buffer, and then stained with 5μl of FITC/Annexin V and 5μl of PI at room temperature for 15 minutes before analyzed by flow cytometry. Fractions of viable, pre-apoptotic, apoptotic and necrosis were analyzed using FlowJo software (TreeStar).

### DNA Extraction and TP53 mutation analysis

Freshly cultured cells (5×106) were harvested and washed with 1x PBS. The genomic DNA was extracted using QIAamp DNA Mini & Blood Mini Kit (Qiagen, Valencia, CA). PCR amplification was performed in a 10μl volume containing 15ng of DNA, 0.25μM of each primer (forward and reverse), 4μl 2.5X LightScanner Master Mix with LCGreen Plus Dye, and 0.5μl 100% DMSO (Sigma) and nuclease free water and followed the protocol (Idaho Technology, Salt Lake City, UT). High Resolution Melting analysis was performed on a LightScanner HR 96 (Idaho Technology). The amplicons were melted from 77°C to 96°C with a heating rate of 0.1°C per second. The data was analyzed using the LightScanner software provided by Idaho Technology. For bi-directional Sanger sequencing, the HRM amplicons were purified using QIAquick PCR Purification Kit (Qiagen, Valencia, CA) and performed as described previously [20].

### Statistics

All results were expressed as means ± SD unless stated otherwise. The unpaired Student's t test was used to evaluate the significance of differences between groups, accepting *p* < 0.05 as level of significance.
